# Evaluation of the Influence of Bottle Type on the Acquisition of SORS Spectra of Extra Virgin and Virgin Olive Oils

**DOI:** 10.3390/foods15030521

**Published:** 2026-02-02

**Authors:** Guillermo Jiménez-Hernández, Fidel Ortega-Gavilán, M. Gracia Bagur-González, Jaime García-Mena, Sandra Montoro-Alonso, Antonio González-Casado

**Affiliations:** 1 Department of Analytical Chemistry, Faculty of Science, University of Granada, C/Fuentenueva w/n, E-18071 Granada, Spain; fog@ugr.es (F.O.-G.); mgbagur@ugr.es (M.G.B.-G.); 2 Animal Health Central Laboratory (LCSA), Department of Chemical Analysis of Residues, Ministry of Agriculture, Fisheries and Food, Camino del Jau w/n, E-18320 Granada, Spain; 3 Department of Chemical Engineering, Faculty of Science, University of Granada, C/Fuentenueva w/n, E-18071 Granada, Spain; jgmena@aceitesmaeva.com (J.G.-M.); smontoro@aceitesmaeva.com (S.M.-A.); 4 Torres Morente S.A.U, CITAI, Avda. Incar 8, E-18130 Granada, Spain

**Keywords:** portable analyzer SORS, extra virgin olive oil, virgin olive oil, similarity index, WSI

## Abstract

The objective of this study was to evaluate the impact of the material (plastic or glass) and color (green or colorless) of extra virgin olive oil (EVOO) and virgin olive oil (VOO) bottles on the acquisition of SORS spectra using portable equipment. Sixteen bottles of EVOO and three bottles of VOO were analyzed, including different volumes. A range of similarity indices was calculated between vial-reference (offline measurements) and bottles (online measurements), including R^2^, COS θ, NEAR, and a new index called WSI (Weighted Similarity Index). WSI is calculated from the pondered linear combination of the previous three, and a threshold of >0.95 is established as high similarity. The results showed that plastic bottles, regardless of color and volume, and colorless glass bottles had WSI values > 0.95. In contrast, green glass bottles demonstrated a lower degree of similarity (WSI < 0.95), which impacted the reliability of their spectral fingerprints. A hierarchical cluster analysis (HCA) was performed by locating EVOO bottles according to their material in two clusters. A study of storage under optimal, non-optimal, and commercial conditions showed that both EVOO and VOO maintain highly similar spectral profiles for 10–18 days (WSI > 0.965), even in bottles purchased in supermarkets. These results demonstrate that the SORS technique is suitable for the direct analysis of olive oils in plastic and colorless glass containers, without the need to open the bottles. The SORS technique is a fast, reliable, non-invasive, and non-destructive tool for quality control of olive oil.

## 1. Introduction

Olive oil, obtained from the fruit of the olive tree (Olea europaea s.p.) [1], is one of the most highly valued and widely consumed food products worldwide. Quality control, authentication, and fraud prevention are therefore key issues in this olive oil sector, as this product is distinguished by its exceptional organoleptic properties and its well-established health benefits, as confirmed by the European Food Safety Authority (EFSA) [2]. Among the different categories of olive oil, virgin olive oils, including extra virgin olive oil (EVOO) and virgin olive oil (VOO), are the most highly prized for their superior quality. In contrast to other edible vegetable oils, they are exclusively obtained through mechanical processes, without the use of chemical solvents. This more natural method of production is also more expensive, resulting in a higher final retail price, especially for EVOO [3,4].

In the scientific field, numerous studies have addressed relevant topics related to virgin olive oil and have traditionally used chromatographic techniques such as High-Performance Liquid Chromatography (HPLC) [5,6,7,8,9] or Gas Chromatography (GC) [10,11,12,13,14] with different detectors. Although these techniques have provided great results, they are invasive and destructive. Spectroscopic methods, such as Near-Infrared (NIR) [15,16,17] or Raman [18,19,20], have also been applied, as they can be non-destructive and/or non-invasive, although these methods generally require the opening of the container and the extraction of an aliquot of the sample. In this context, spatially offset Raman spectroscopy (SORS) represents a promising alternative, as it allows direct measurement through the original olive oil containers without the need to open them. This is made possible by the displacement between the point of incidence of the excitation laser and the signal collection point (offset) [21,22,23]. Furthermore, as it does not require sample preparation or the use of reagents or solvents, this technique is in line with the principles of green analytical chemistry [24]. Although SORS-based portable analyzers have been used most frequently in sectors such as pharmaceuticals [22,25], there are also documented applications in the food sector. The literature describes studies about certain problems in specific products, including meat [26], cheese [27,28], butter [29], tomatoes [30], and alcoholic beverages, including spirits [31,32] and white wine [33].

In particular, the use of the SORS technique in olive oils has become increasingly important in recent years. It has been applied in the authentication of EVOO [2], the discrimination/classification of vegetable oils [34], the detection of adulteration with sunflower oil [35] and the development of sustainable strategies for the authentication of olive oil [36]. However, it should be noted that only the studies by Varnasseri [2,35], Jiménez-Hernández and Horns [35] used commercial packaging, albeit with significant limitations. The first study was constrained to a singular variety of commercial clear glass bottles, characterized by a small capacity of 125 mL. Similarly, the second study utilized a commercial polyethylene terephthalate (PET) bottle (RAMAPET N180) with a capacity of 1 L, including not only its grammage of 23.5 g and its thickness of 0.2 ± 0.02 mm, but also its technical data. However, in both cases [2,35], the objective was not to evaluate different types of commercial bottles. In contrast, the third study [35] reported technical difficulties arising from the lack of adaptation of the SORS equipment to certain bottle formats due to the absence of specific adapters.

To authors’ knowledge, no study analyzed virgin olive oil using a portable SORS-based spectrometer directly through a considerable number of commercial bottles of different materials and colors, without the need to open them. We adapted to all packaging formats with specific parts without loss of laser efficiency and placed the analyzer in the correct position without any gap between it and the wall of the bottles. We included the technical data sheets of all the packaging used, provided by a leading company in the olive oil sector (Torres Morente S.A.U.) (see in SM the technical data sheets 1–8, which include Tables S1–S13 and Figures S1–S8). This aspect reinforces the reproducibility of the results and broadens the practical applicability of the SORS technique in real scenarios of quality control and authentication of olive oils.

The use of a portable SORS spectrometer allows direct measurement through closed commercial containers and provides operational and environmental advantages for the olive oil sector: it reduces the consumption of solvents and reagents, shortens analysis times, minimizes the instrumentation and resources required, and enables measurements to be taken on site. Therefore, this study aimed to use a portable SORS-based analyzer to evaluate the similarity between the instrumental SORS fingerprint obtained in a standardized vial (offline measurements) and the instrumental SORS fingerprint recorded through direct measurements through commercial EVOO and VOO bottles (online measurements), including variations in the material and color of the bottles. To this end, the similarity indices COS (cosine of the angle between two vectors), R^2^ (coefficient of determination), and NEAR (proximity based on Euclidean distance in an orthogonal space) were calculated. A new similarity index, WSI (Weighted Similarity Index), is also proposed, with the aim of integrating these metrics into a single measure of concordance. This approach offers a non-invasive, non-destructive, sustainable, and applicable alternative for quality control and authentication of olive oils. In instances where commercial bottles exceed the similarity criterion, this proposed methodology could have a direct impact on the olive oil sector, as it would save time and money and implement a sustainable methodology.

## 2. Materials and Methods

### 2.1. Olive Oil Bank Samples

Two coupage categories of olive oil (EVOO and VOO, from blending of different olive varieties), constituting two different commercial batches, i.e., EVOO and VOO batches, were used in this study. Thus, an EVOO sample bank containing 16 bottles, with 14 of them from different types of package materials, one as a duplicate of one package material, all of them obtained directly from the oil packaging company (Torres Morente S.A.U, Granada, Spain). Finally, a new duplicate of the package material that had been selected previously was purchased from a local supermarket.

The VOO data bank, constituted by only three bottles packed with the same material, was used to study the influence of time and storage conditions.

All the samples coming from the oil packaging plant were analyzed immediately after their bottling.

### 2.2. Types of Bottle

**Figure 1:**
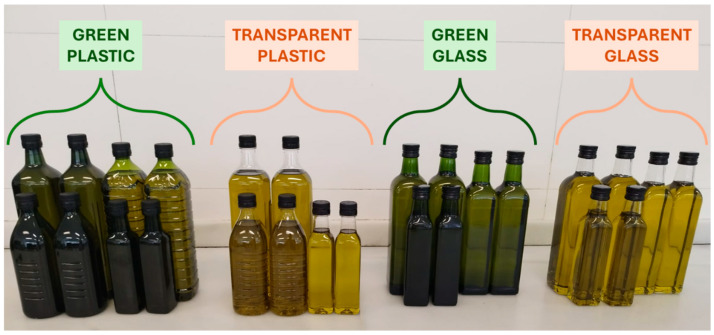
Visualization of the different bottles used for this study.

Bottles ranging from 250 mL to 5 L were used in this study. They were fabricated using two different materials (plastic and glass), both in two colors (colorless and green) (see Figure 1). All bottles were supplied by Torres Morente S.A.U, accompanied by their technical data sheets (see in SM the technical data sheets 1–8, which include SM Tables S1–S13 and SM Figures S1–S8).

### 2.3. SORS Measurement

A portable spectrometer, the Vaya Raman model (Agilent Technologies, Santa Clara, CA, USA), was used based on the SORS technique. This device allows for measurements directly through some types of containers [37] without either sample destruction or invasion. The spectrometer is a lightweight (1.86 kg), small (257 × 127 × 60 mm), compact, and portable device with approximately four hours of autonomy. It also has a wide operating temperature range of −5 °C to 35 °C and can function in up to 95% relative humidity without condensation.

The spectral range of the equipment covers 350 to 2000 cm^−1^, with a resolution of 12–20 cm^−1^ and spectral stability of less than 1.5 cm^−1^ over 24 h, in accordance with ASTM E2529-06 [38]. The analyzer is equipped with a Class 3B laser (450 mW) and operates at a wavelength of 830 nm. For spectral acquisition, Vaya Raman uses a cooled Charge-Coupled Device (CCD) optimized for the NIR spectra zone, which employs a total acquisition time per sample time of less than 2 min.

To obtain SORS spectra, the instrument software (Vaya Software, version 1.2.221) performs a pre-processing procedure consisting of (i) eliminating the contribution of the outermost layers of the container, (ii) adjusting the baseline of the recorded spectrum, and (iii) normalizing the intensity of the SORS spectrum from zero to one. Finally, the SORS spectrum of each sample is obtained by scaled subtraction of two spectra: (i) the zero-shift spectrum, dominated by the Raman contribution from the vial/container, collected at the same location as the laser illumination, and (ii) the spatial shift spectrum, enriched with information from the subsurface sample, acquired by shifting the laser position to the signal collection point (set at 0.6 mm). Thus, the acquisition of SORS spectra through an original container could be possible due to the capability of the equipment to minimize interference from the container material [23,39]. The final SORS spectra were recorded in.csv format and exported to MATLAB (version 9.3, Mathworks Inc., Natick, MA, USA) for subsequent analysis. For similarity studies, no additional pre-processing was applied before the calculation of the used indices to warrant that no artificial artifacts would be included.

Given the objective of this research, which is to study the possibility of using the Raman spectrum of a real sample of EVOO and VOO measured directly through its original packaging, EVOO and VOO were directly analyzed directly taken an aliquot from the oil coupage tank and transferred to borosilicate vial (4 mL Raman vials with a thickness of 1 mm) taken these spectra as the reference spectrum for each oil. This choice is attributable to the negligible or even non-existent contribution of the vial to the final spectrum. Each vial was cleaned and placed in the instrument sample holder, which as opaque permit to record the spectrum in the absence of any influence of the environmental radiation. To minimize the variability, three spectra were recorded, rotating the vial between each measurement. These spectra were used to obtain the average spectrum of each type of oil used as a reference, not only to evaluate the viability of the container to obtain the oil spectrum from different kinds of bottles studied, but also to indirectly study the preservation of each oil in the different conservation conditions of the samples.

**Figure 2:**
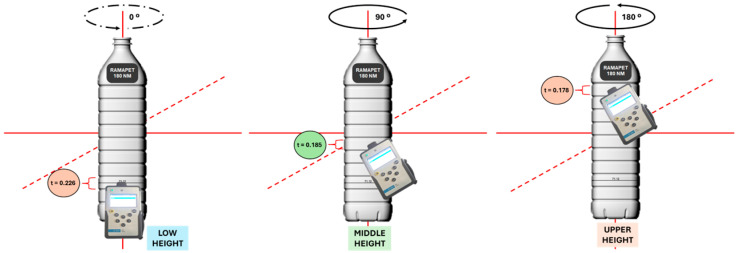
Flowchart for taking direct measurements with the portable SORS analyzer through olive oil bottles without opening them. The bottle used in this example is the RAMAPET N180. t: Thickness.

The measurements throughout the bottles were carried out using specific adapters supplied by the manufacturer (see Figure S9) [37]. In all the cases, the spectrometer was positioned over the bottle as can be seen in Figure 2, taking the measures at three heights, lower, middle, and upper and keeping the bottle in a vertical position, to consider the non-uniform thickness of plastic and glass bottles (see SM Tables S5, S7, S9, S11 and S13). It is noteworthy that the laser was applied to flat parts of the bottle and avoided the label, thereby ensuring that neither the corrugated parts nor the conic zones proximally to the bottleneck were focused on. Finally, for each material and color, an average spectrum obtained by combining the spectra at different heights was considered as the representative spectrum of the bottle.

All measurements, both in vials and bottles, were carried out at room temperature (25 °C). Furthermore, for direct measurements in each bottle, as there was no equivalent opaque accessory available, the recordings were made in complete darkness, without natural or artificial light.

### 2.4. Study of Similarity in EVOO and VOO Preservation

In both cases, three bottles of each type of oil were available, subjected to different storage conditions: (i) optimal conditions, characterized by absence of light and cold storage, (ii) non-optimal conditions, consisting of storage at room temperature and exposure to natural light, and (iii) one bottle purchased directly from a local supermarket, representative of marketed conditions.

Since the bottle purchased in the supermarket had a screen-printed commercial label, the other two bottles in the study were labelled with the same commercial label, placed in the same position, to ensure homogeneous experimental conditions. The measurement procedure was identical to that described in Figure 2, with the additional precaution of ensuring that the excitation laser did not pass through the commercial label. This was implemented to avoid any interference in the acquisition of the SORS spectra.

### 2.5. Similarity Indices

To compare the instrumental Raman fingerprints obtained from the EVOO vial with the corresponding SORS fingerprints from the original bottles filled with the same samples, three similarity indices were estimated as follows:

(i) R^2^ (coefficient of determination), i.e., the square of the Pearson correlation coefficient between two data vectors (spectra obtained for each container vs. reference EVOO/VOO spectrum, represented in general terms as Y_A_ and Y_B,_ respectively), defined in Equation (1), and it takes values from zero to one [40]:

(1) $$R^{2}\left(Y_{A},Y_{B}\right)=\frac{{\left[\sum \left(\left(Y_{Ai}-{\bar{Y}}_{A}\right)\cdot \left(Y_{Bi}-{\bar{Y}}_{B}\right)\right)\right]}^{2}}{\sum {\left(Y_{Ai}-{\bar{Y}}_{A}\right)}^{2}\cdot \sum {\left(Y_{Bi}-{\bar{Y}}_{B}\right)}^{2}}$$

(ii) COS θ (cosine of the angle) obtained by Equation (2) [41]. The COS θ will be one when the angle is 0°, i.e., the two spectra have the same orientation, i.e., they are similar:
  (2) $$COS\;\theta \;\left(Y_{A},Y_{B}\right)=\frac{\sum (Y_{Ai}\cdot Y_{Bi})}{\sqrt{\sum {Y_{Ai}}^{2}\cdot \sum {Y_{Bi}}^{2}}}$$

(iii) NEAR (nearness index, Equation (3)), is a measurement of the normalized Euclidean distance between the considered spectra obtained for each container and reference EVOO/VOO spectrum [42], and it is used to describe the proximity between each spectrum, varying from zero to one:
  (3) $$NEAR\left(Y_{A},Y_{B}\right)=1-\sqrt{\frac{\sum (Y_{Ai}-Y_{Bi}{)}^{2}}{\sum (Y_{Ai}+Y_{Bi}{)}^{2}}\;}$$

These indices provide information about similarity between two data vectors at a different sensibility level, being NEAR the most restrictive one. This is because both R^2^ and cos θ ensure the similarity between spectral profiles, exhibiting reduced sensitivity in comparison to NEAR, which considers the proximity between the profiles under comparison and is consequently more influenced by the background signal. Considering this fact, a new similarity index, denoted as Weighted Similarity Index (WSI), has been proposed as a pondered linear combination of R^2^, cos θ, and NEAR, as can be seen in Equation (4).

(4) $$WSI=0.15\times COS\;\theta \;(Y_{A},Y_{B})+0.30\times R^{2}(Y_{A},Y_{B})+0.55\times NEAR(Y_{A},Y_{B})$$

## 3. Results and Discussion

To study the feasibility of measuring the Raman spectrum of EVOO and VOO samples through each commercial bottle, the similarity indices (Equations (1)–(4)) were estimated. In all cases, a threshold value to consider this feasibility was established based on the similarity between the SORS fingerprints obtained for each container and the reference EVOO/VOO fingerprints. Thus, the respective spectra were considered highly similar when the similarity indices values were >0.95, with a reliable similarity when the indices values were ranged between 0.90 and 0.95, and without similarity when the indices took values lower than 0.90.

### 3.1. Study of the Influence of Bottling EVOO in Plastic and Glass Bottles

#### 3.1.1. Raman-SORS Fingerprints Comparison

**Figure 3:**
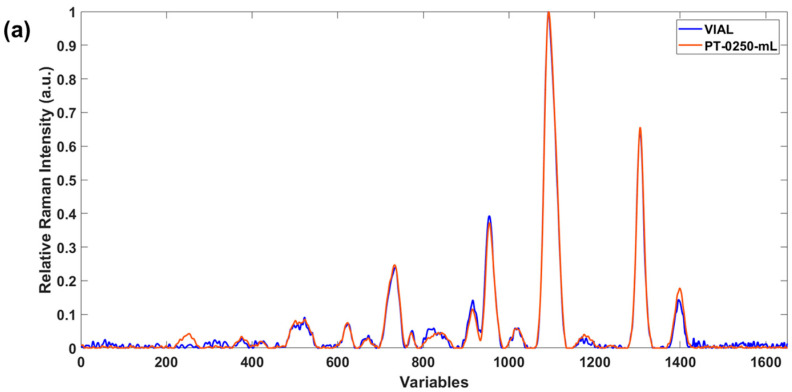

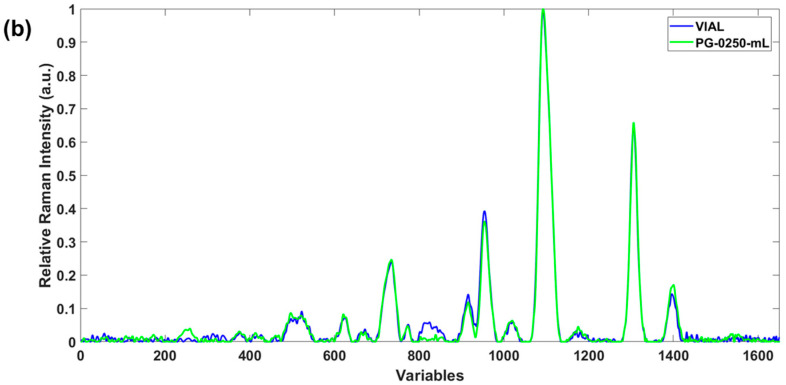
Comparison between the fingerprints on the vial and (**a**) plastic transparent bottle and (**b**) green plastic bottle.

**Figure 4:**
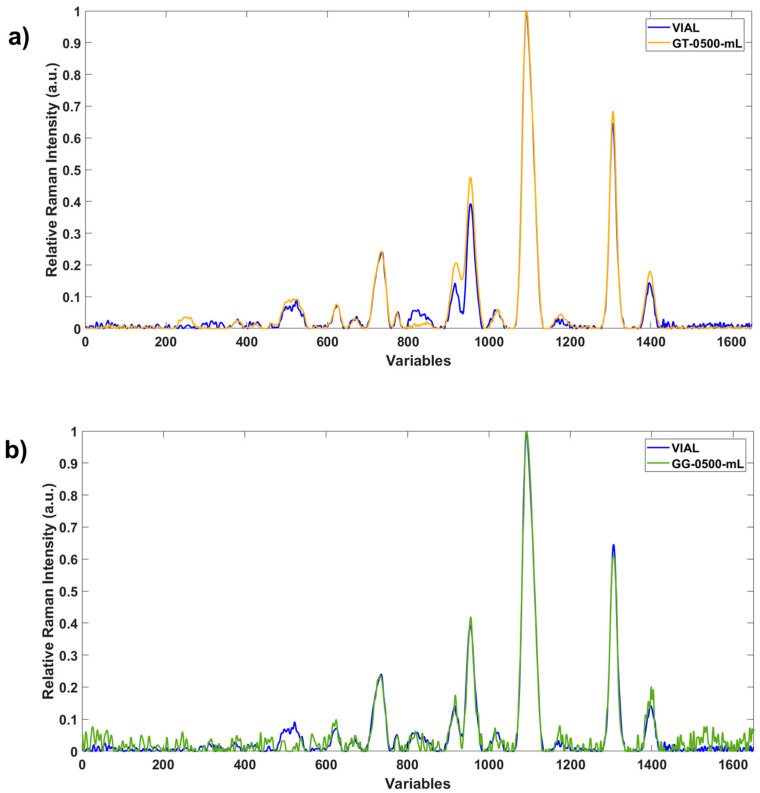
Comparison between the fingerprints on the vial and (**a**) a glass transparent bottle and (**b**) a green glass bottle.

Figure 3 and Figure 4 show the superimposed SORS fingerprints of the EVOO sample (bottled in plastic and glass, respectively) on the fingerprint of the same oil used as a reference. As can be seen in Figure 3, the EVOO fingerprints are practically equivalent in all the wavenumber range of the spectra excepting the variable range of 800 to 900 (corresponding to wavenumbers among 1150 to 1250 cm^−1^). This region corresponds to twisting trans-alkene and can be explained as an absorption of light from the container, with this phenomenon more intense when the plastic is colored, i.e., disappearing practically.

In glass bottles (Figure 4), the EVOO fingerprints are less equivalent than plastic bottles across much part of the fingerprint. This fact can be attributed not only to the material, i.e., thickness, but also to the color (see Figure 4a,b).

#### 3.1.2. Raman-SORS Fingerprint Similarity Indices

**Figure 5:**
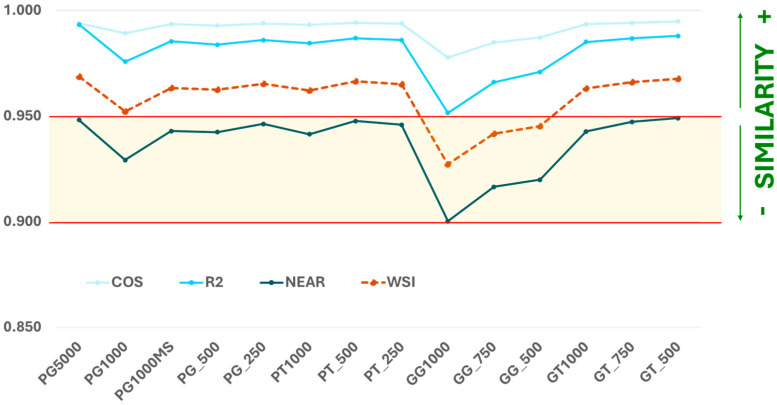
Similarity indices calculated for each type of bottle with respect to the reference. PT: Colorless plastic (technically transparent), PG: Green plastic, MS: Most sale, GT: Colorless glass, and GG: Green glass.

The similarity study through the four similarity indices described in Section 2.4 was made according to the methodology by comparison of the average fingerprint of each type of bottle (material, color, and volume) and the EVOO reference fingerprint. Figure 5 and Table S14 show the values obtained for each similarity index in all the cases. The COS θ index yielded the highest similarity values, whereas the R^2^ index showed slightly greater differentiation between samples. In contrast, the NEAR index produced values consistently below 0.95, which justifies the introduction of the WSI parameter. Combining COS θ, R^2^, and NEAR using a weighted linear combination would minimize the limitations of the NEAR index and provide a more balanced assessment of SORS fingerprints’ similarity.

According to Figure 5, the plastic bottles (colorless and green) with different volumes (1000, 500, and 250 mL formats in both cases) and an additional 5000 mL format for plastic green, showed a high fingerprint similarity compared to the EVOO reference fingerprint measured, not only for PT and PG types but also for GT types, since COS θ, R^2^, and WSI indices are enclosed in the threshold high similarity area. The last one, WSI, permits avoiding the high sensitivity shown for the NEAR index, as was quoted previously. Therefore, the SORS spectra are practically equal whether the bottles or the vials are used. This fact confirms that these types of containers are adequate to record the SORS spectrum as the vial is used.

For glass bottles (colorless and green) with different volumes (1000, 750, and 500 mL formats), different behaviour was observed between colorless and green glasses. Thus, the colorless glass bottles (GT) yielded results comparable to those obtained for plastic containers, showing a high SORS fingerprint similarity with the EVOO reference fingerprint (because COS θ, R^2^, and WSI indices are also enclosed inside the threshold high similarity area). In contrast, the green glass bottles (GG) present WSI values below 0.95 regardless of the capacity of the bottles. This fact might indicate a lower degree of reliable similarity with respect to the measurement through the vial.

#### 3.1.3. Influence of Technical Properties on SORS Fingerprints

To evaluate the influence of volume and thickness and using the WSI index as a new variable associated with fingerprints, a Hierarchical Cluster Analysis (HCA) was performed using Ward’s method and the Euclidean distance. The analysis was conducted with Statgraphics 19™ (Statgraphics Technologies, Inc., The Plains, VA, USA). The data matrix consisted of the 14 types of bottles analyzed—corresponding to plastic and glass containers, both green and colorless (this information was included in the code of the sample)—and three variables previously commented on (volume, thickness, and WSI values), resulting in a 14 × 3 matrix. The number of clusters was selected, attending to a D_linkage_ = 2/3 of D_max_ as an internal criterion.

**Figure 6:**
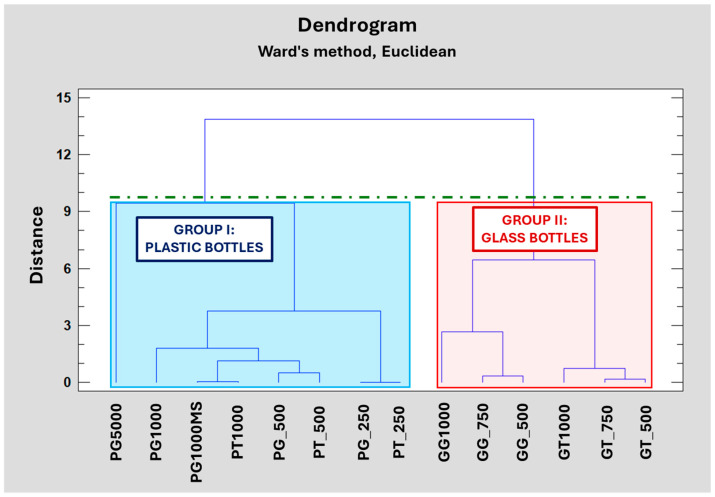
Dendrogram obtained from the HCA analysis. The green dotted line represents the D_Linkage_ (D_Linkage_ = 2/3 D_max_) used for the assignment of natural grouping.

Figure 6 shows the dendrogram clearly differentiates between two clusters based on container material: Group I consists of plastic bottles, while Group II consists of glass bottles. This fact might be justified by the thickness of the material used in the elaboration of the bottles, given that plastic bottles exhibit lower thickness than glass bottles.

In Group I, the 5 L format is highlighted, as it is distinctly different from the other bottles in this group, which were grouped based on bottle volume independently of the color of the plastic. Whereas Group II was formed based on the color of the packaging, and was not influenced by the volume.

### 3.2. Influence of Time and Storage Conditions on EVOO/VOO Fingerprints and Their Similarity

**Figure 7:**
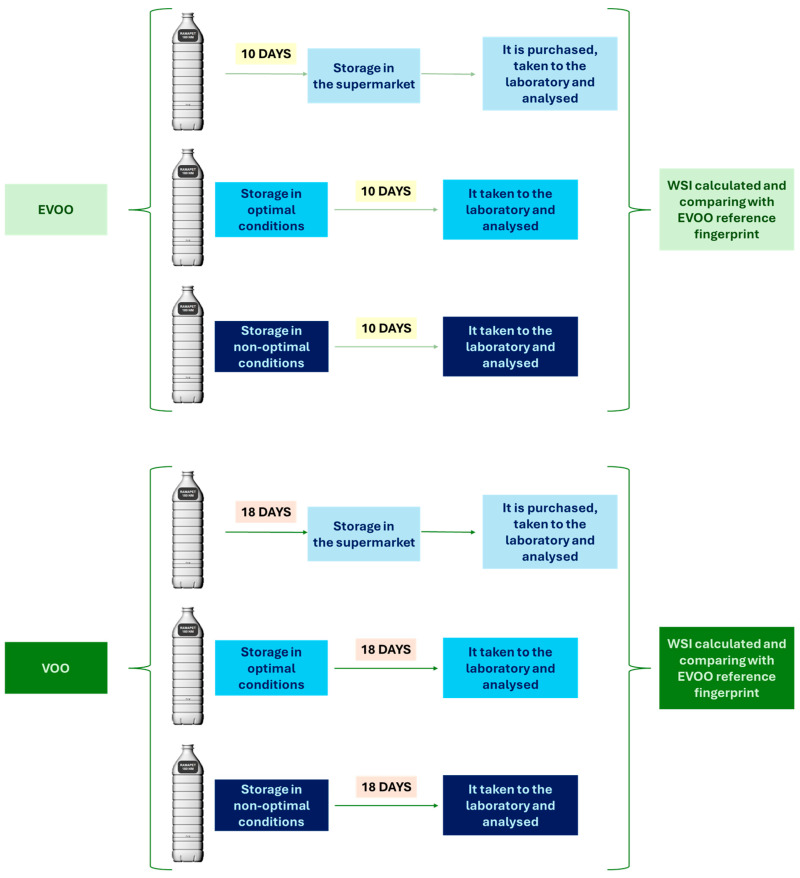
Scheme for analyzing the influence of time and storage conditions on EVOO and VOO samples.

After the verification of the feasibility to utilize the container, mainly colorless and green plastic bottles, directly to obtain the SORS spectrum of EVOO, the potential influence of two factors deemed to be significant was evaluated: the elapsed time since packaging and the packaging conditions on the subsequent recording of the EVOO/VOO SORS spectra through the bottles. Figure 7 shows the procedure followed to evaluate this influence, based on the use of a green plastic bottle (PG_1000_MS) commonly used by the packaging company (mainly due to the green color better preserving the composition of the EVOO and VOO during storage) and consequently distributed to points of sale. Table 1 shows the obtained results, including COS θ, R^2^, and NEAR, needed to calculate the WSI index.

For both EVOO and VOO bottles, as shown in Table 1, the proposed WSI index values indicated that independently of the three conditions used (optimal, non-optimal, and stored for sale), there was a high degree of SORS fingerprint similarity after 10 and 18 days for EVOO and VOO, respectively, with values exceeding the threshold in all samples, arising 0.965 for both EVOO and VOO, being even possible to consider it constant for EVOO bottles samples.

Furthermore, the COS θ index consistently yielded the highest similarity values, reaching at least 0.993 in all cases. The R^2^ index also showed high values, particularly for the optimal and non-optimal conditions considered, although comparable values were observed under the supermarket conditions. The NEAR index exhibited the lowest similarity levels, with more favourable values for the well-preserved samples, followed by those stored under non-optimal conditions, and the lowest values corresponding to the bottles purchased from retail shelves.

These results suggest that EVOO and VOO stored under optimal conditions better preserved their original spectral fingerprints, whereas progressively lower spectral similarity was observed for samples subjected to non-optimal storage and retail exposure. It is evident that, despite the measurements being performed under analytical conditions completely different from those at the time of packaging, the similarity metrics vary only slightly.

## 4. Conclusions

The combination of a portable SORS analyzer with instrumental fingerprint similarity measurements, such as the one proposed here, facilitates the acquisition of SORS spectra of extra virgin and virgin olive oil samples directly from their container, i.e., the bottle.

Regarding the defined similarity index (WSI), by performing a linear weighting of indices commonly used in this type of study, such as COS, R^2^, and NEAR, it is possible to minimize the high sensitivity of the latter by balancing the correlation, the instrumental fingerprint profile, and the distance between the observed instrumental fingerprints.

The results show that, while the capacity of the containers and the signal collection area have less influence, the most important factors for spectral fingerprint quality are the material of the containers (plastic or glass) and especially their color (colorless or green). It was determined that all containers analyzed are suitable for direct measurement, except for green glass bottles, which do not meet the required spectral fingerprint similarity criteria (WSI > 0.95). This suggests that, before taking measurements through the bottle, it would be necessary to verify by similarity studies whether the bottle’s material affects the obtained SORS fingerprint. The HCA analysis demonstrates that the packaging material is the predominant factor in differentiating SORS instrumental fingerprints, which is attributable to the thinner thickness of plastic bottles, whose cluster is mainly influenced by volume. In glass containers, the formation of subgroups is determined by coloring, regardless of volume.

Considerations offer a good opportunity for the implementation of a portable SORS analyzer as a viable tool in the food industry, which would permit its incorporation into quality control procedures with an indirect impact on the benefit to consumer health, safety and without an important environmental cost. Thus, the portable SORS device represents a sustainable, viable, and rapid alternative, enabling measurements without the need to open the bottles or manipulate their contents.

## Figures and Tables

**Table 1:** Average similarity index values for the EVOO and VOO bottles under study in good condition, in poor condition, and stored for sale.

Commercial Category	Bottle Code	COS θ	R^2^	NEAR	WSI
EVOO	Op ^1^. Cond ^2^.	0.994	0.987	0.950	0.968
	Non-Op. Cond.	0.994	0.987	**0.949**	0.967
	Supermarket	0.994	0.985	**0.946**	0.965
VOO	Op. Cond.	0.996	0.991	0.961	0.975
	Non-Op. Cond.	0.995	0.988	0.955	0.971
	Supermarket	0.993	0.985	**0.946**	0.965

Note: Values of similarity indices lower than the 0.95 threshold are highlighted in bold red. ^1^ Op: Optimal, and ^2^ Cond: Conditions.

## Data Availability

The original contributions of this study are presented in this article/Supplementary Materials. Further inquiries can be directed to the corresponding authors.

## Supplementary Materials

The following supporting information can be downloaded at: https://www.mdpi.com/article/10.3390/foods15030521/s1, Figure S1: Design for 750 mL capacity glass bottles; Figure S2: Design for 500 mL capacity glass bottles; Figure S3: Design for 250 mL capacity glass bottles; Figure S4: Design for 5000 mL capacity plastic bottles; Figure S5: Design for 1000 mL capacity plastic bottles known as most sale bottle; Figure S6: Design for 1000 mL capacity plastic bottles; Figure S7: Design for 500 mL capacity plastic bottles; Figure S8: Design for 250 mL capacity plastic bottles; Figure S9: Appreciation of how the specific adapter for the Vaya Raman portable device fits onto the surface of the bottle; Table S1: Characteristics of the 750 mL glass bottle; Table S2: Characteristics of the 500 mL glass bottle; Table S3: Characteristics of the 250 mL glass bottle; Table S4: Characteristics of the 5000 mL plastic bottle; Table S5: Thickness of the 5000 mL plastic bottle; Table S6: Characteristics of the 1000 mL plastic bottle known as most sale bottle; Table S7: Thickness of the 1000 mL plastic bottle known as most sale bottle; Table S8: Characteristics of the 1000 mL plastic bottle; Table S9: Thickness of the 1000 mL plastic bottle; Table S10: Characteristics of the 500 mL plastic bottle; Table S11: thickness of the 500 mL plastic bottle; Table S12: Characteristics of the 250 mL plastic bottle; Table S13: Thickness of the 250 mL plastic bottle; Table S14: Average similarity index values for plastic containers.
