# Supplementary material for: Evaluation of the Influence of Bottle Type on the Acquisition of SORS Spectra of Extra Virgin and Virgin Olive Oils
Source: Foods. 2026 Feb 2;15(3):521. doi: 10.3390/foods15030521 (PMC12897468; doi:10.3390/foods15030521)
Supplement: Supplementary file 1 [file foods-15-00521-s001.zip › foods-4074248-supplementary.pdf]

Research Article

# Evaluation of the Influence of Bottle Type on the Acquisition of SORS Spectra of Extra Virgin and Virgin Olive Oils

Jiménez-Hernández, G.[1]✉; Ortega-Gavilán, F.[1,2]; Bagur-González, M.G.[1]; García-Mena, J.[3,4] Montoro-Alonso, S.[4] & González-Casado, A.[1]✉

*1 Department of Analytical Chemistry, Faculty of Science, University of Granada, C/ Fuentenueva w/n, E-18071, Granada (Spain)*

*2 Department of Chemical Analysis of Residues, Animal Health Central Laboratory (LCSA), Ministry of Agriculture, Fisheries and Food, Camino del Jau w/n, E-18320, Santa Fe (Granada)*

*3 Department of Chemical Engineering, Faculty of Science, University of Granada, C/ Fuentenueva w/n, E-18071, Granada (Spain)*

*4 Torres Morente S.A.U, CITAI, Avda. Incar 8, 18130, Escúzar (Granada)*

✉ Corresponding author, e-mail: guillermojimenez@ugr.es; Phone: +34 958240797.
✉ Corresponding author, e-mail: agcasado@ugr.es Phone: +34 958240799.

GRUPO MAEVA TORRES-MORENTE
Av. Íncar 8, CITAI
18130 Escúzar - Granada
Tel: 958 99 10 33
www.aceitesmaeva.com

The data below has been extracted from the packaging technical sheet provided by our supplier. For reasons of industrial property, the manufacturer's details are not disclosed.

## TECHNICAL DATA SHEETS FROM OLIVE OIL BOTTLES

## TECHNICAL DATA SHEET 1

- **MARASCA GLASS BOTLLE 750 mL**

**Table S1.** Characteristics of the 750 mL glass bottle.

| Attribute | Details |
|---|---|
| Approx. Weight | 500 g |
| Total Height | 276.5 + 1.7 mm |
| Capacity / Body Diameter | At lip level: 775 cc / 10.0 mm; At 55 mm from lip: 753 cc / 110.0 mm |
| Labeling Zone | 37.5 ± 1.3 mm |
| Label Guard | 58.1 ± 1.3 mm |
| Mouth Features / Model | 490 - Mouth BVP 31.5 Std |
| Standard | EN-15543 |
| Plan Number | 6718-1 |
| Tolerances | Ovalization ± 1.3 mm; Verticality relative to axis: 3.1 max; Mouth parallelism: 0.6 max |
| Thermal Shock | 42 °C |
| Mechanical Shock | 35 put/sec min |
| Slide Angle | Between 7° and 25° |
| Pressure Resistance | Internal instant / Internal perm (1'): kg/cm² |
| Refillable Container | No |
| CO2 Volume | 0.0 |
| Standardization | EN-15543 |
| Observations | — |

The glass type is soda-lime glass, with the following composition:

- $SiO_2$: 69% to 74%
- $Na_2O$ + $K_2O$: 12% to 16%
- $CaO$ + $MgO$: 10% to 13%
- $Al_2O_3$ + $Fe_2O_3$ 0.5% to 4.0%

Glass thickness: 1 mm

Green colour in glass is achieved through the incorporation of mineral colorants, primarily chromite or iron oxide compounds.

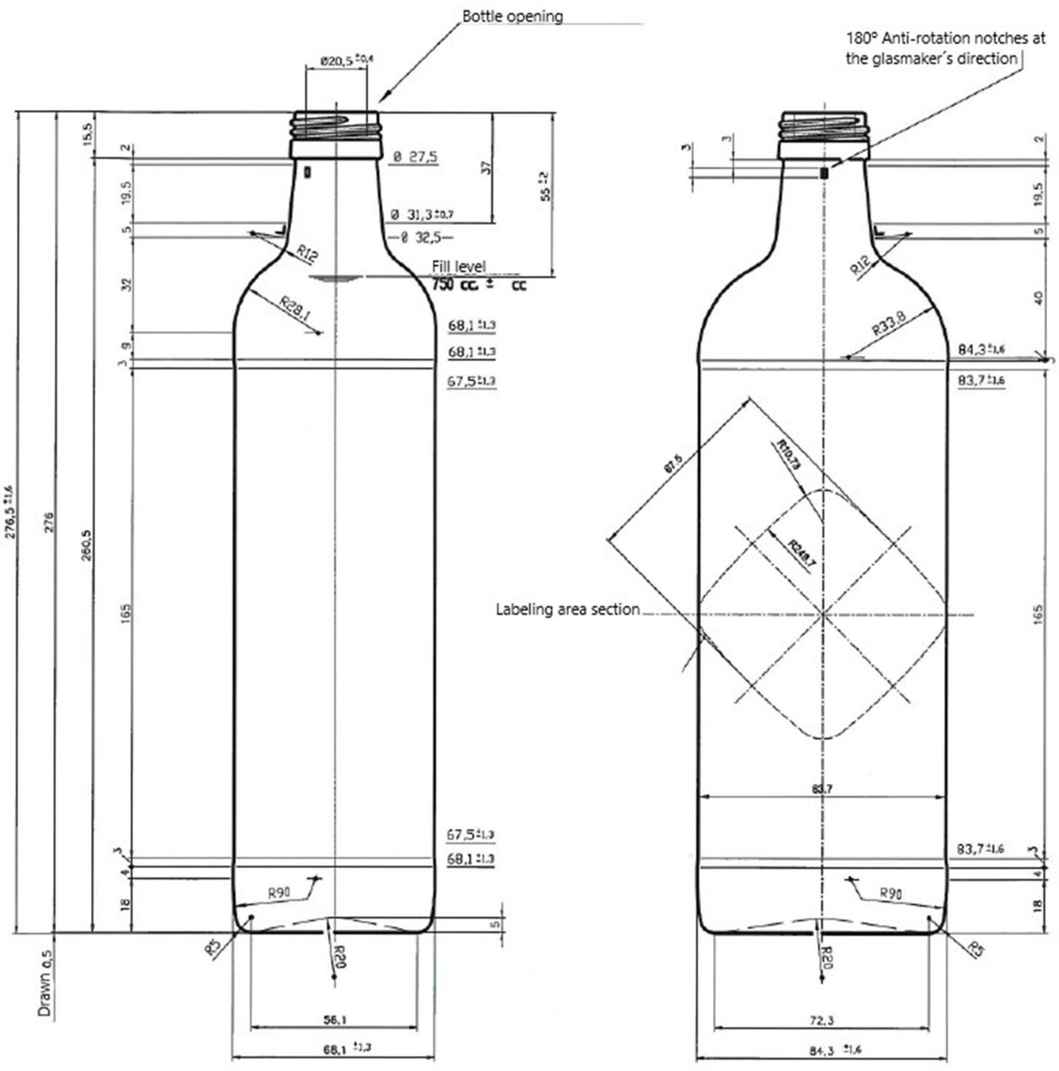


**Figure S1.** Design for 750 mL capacity glass bottles.

# TECHNICAL DATA SHEET 2

- **MARASCA GLASS BOTLLE 500 mL**

**Table S2.** Characteristics of the 500 mL glass bottle.

| Attribute | Details |
|---|---|
| Approx. Weight | 380 g |
| Total Height | 266 mm + 1.7 mm |
| Capacity / Body Diameter | At lip level: 532 cc ±10 cc |
| Labeling Zone | 6.9 mm ±1.2 mm |
| Label Guard | 7.5 mm ±1.2 mm |
| Mouth Features / Model | Model 490-MOUTH BVP 31.5 Std |
| Standard | EN-15543 |
| Plan Number | 6718-1 |
| Tolerances | Ovalization +1.2 mm; Verticality max. 3.0 mm; Mouth parallelism max. 0.6 mm |
| Thermal Shock | 42 °C |
| Mechanical Shock | ≥35 pul/sec |
| Slide Angle | Between 7° and 25° |
| Pressure Resistance | Internal instant / Internal perm (1'): kg/cm² |
| Refillable Container | No |
| CO2 Volume | 0.0 |
| Standardization | EN-15543 |
| Observations | – |

The glass type is soda-lime glass, with the following composition:

- $SiO_2$: 69% to 74%
- $Na_2O$ + $K_2O$: 12% to 16%
- CaO + MgO: 10% to 13%

- $Al_2O_3 + Fe_2O_3$ 0.5% to 4.0%

Glass thickness: 1 mm

Green colour in glass is achieved through the incorporation of mineral colorants, primarily chromite or iron oxide compounds.

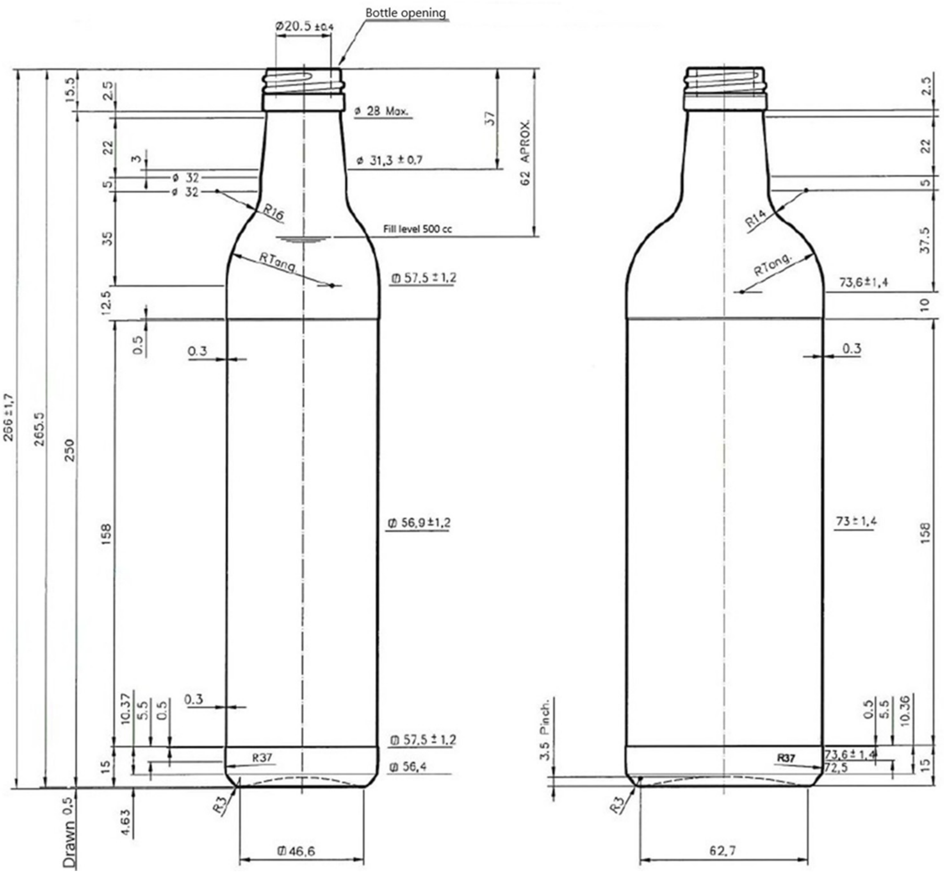


**Figure S2.** Design for 500 mL capacity glass bottles.

# TECHNICAL DATA SHEET 3

- **MARASCA GLASS BOTLLE 250 mL**

**Table S3.** Characteristics of the 250 mL glass bottle.

| Attribute | Details |
|---|---|
| Approx. Weight | 255 g |
| Total Height | 213.0 + 1.5 mm |
| Capacity / Body Diameter | At lip level: 268 cc / 6.0 mm |
| Labeling Zone | 46.6 ± 1.1 mm |
| Mouth Features / Model | 490 - Mouth BVP 31.5 Std |
| Standard | EN-15543 |
| Plan Number | 6718-1 |
| Tolerances | Ovalization ± 1.1 mm; Verticality relative to axis: 2.4 max; Mouth parallelism: 0.6 max |
| Thermal Shock | 42 °C |
| Mechanical Shock | 35 put/sec min |
| Slide Angle | Between 7° and 25° |
| Pressure Resistance | Internal instant / Internal perm (1'): kg/cm² |
| Refillable Container | No |
| CO2 Volume | 0.0 |
| Standardization | EN-15543 |
| Observations | — |

The glass type is soda-lime glass, with the following composition:

- $SiO_2$: 69% to 74%
- $Na_2O + K_2O$: 12% to 16%
- $CaO + MgO$: 10% to 13%
- $Al_2O_3 + Fe_2O_3$ 0.5% to 4.0%

GRUPO MAEVA TORRES-MORENTE
Av. Íncar 8, CITAI
18130 Escúzar - Granada
Tel: 958 99 10 33
www.aceitesmaeva.com

Glass thickness: 1 mm

Green colour in glass is achieved through the incorporation of mineral colorants, primarily chromite or iron oxide compounds.

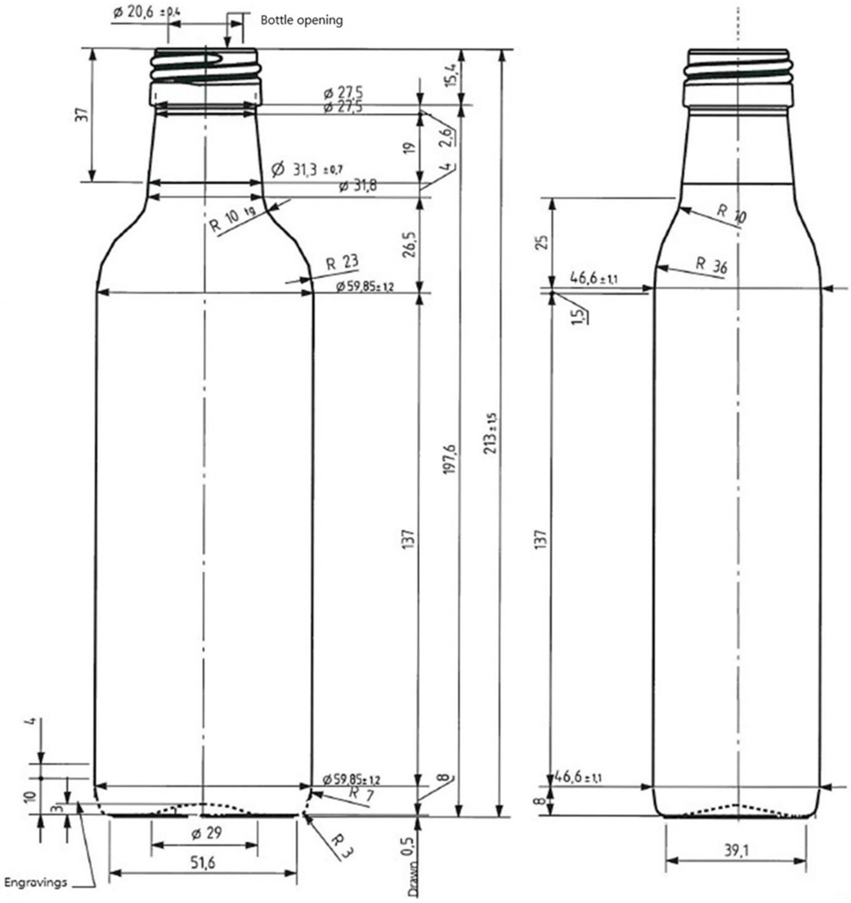


**Figure S3.** Design for 250 mL capacity glass bottles.

GRUPO MAEVA TORRES-MORENTE
Av. Íncar 8, CITAI
18130 Escúzar - Granada
Tel: 958 99 10 33
www.aceitesmaeva.com

# TECHNICAL DATA SHEET 4

- **SQUARE PET BOTTLE 5 L**

**Table S4.** Characteristics of the 5000 mL plastic bottle.

| Attribute | Details |
|---|---|
| Material | PET |
| Filling point capacity | 5000 ± 10 mL |
| Overfill | 5050 ± 10 mm |
| Filling point | Mm |
| Weight | 75 ± 0.5 gr |
| Vertical load | ≥ 20 kg |
| Minimum wall thickness | 0.2 mm |
| **Dimensions** | |
| Height | 347.5 ± 1.5 mm |
| Width | 132.5 ± 1 mm |
| Depth / bottom | 150 ± 1 mm |

**Table S5.** Thickness of the 5000 mL plastic bottle.

| THICKNESS | | | |
|---|---|---|---|
| | MOLD 1 | | |
| | Measurement (mm) | SIDE A | SIDE B |
| 12 | 314 | 0,523 | 0,488 |
| 11 | 290 | 0,370 | 0,348 |
| 10 | 262 | 0,318 | 0,307 |
| 9 | 234 | 0,282 | 0,274 |
| 8 | 206 | 0,274 | 0,262 |
| 7 | 181 | 0,262 | 0,257 |
| 6 | 156 | 0,269 | 0,239 |
| 5 | 130 | 0,257 | 0,246 |
| 4 | 105 | 0,287 | 0,244 |
| 3 | 81 | 0,267 | 0,241 |
| 2 | 54 | 0,279 | 0,249 |
| 1 | 30 | 0,264 | 0,267 |

The color is provided through PET color additives, which are mixed with the resin during the preform manufacturing process. Both transparent and green PET have been used.

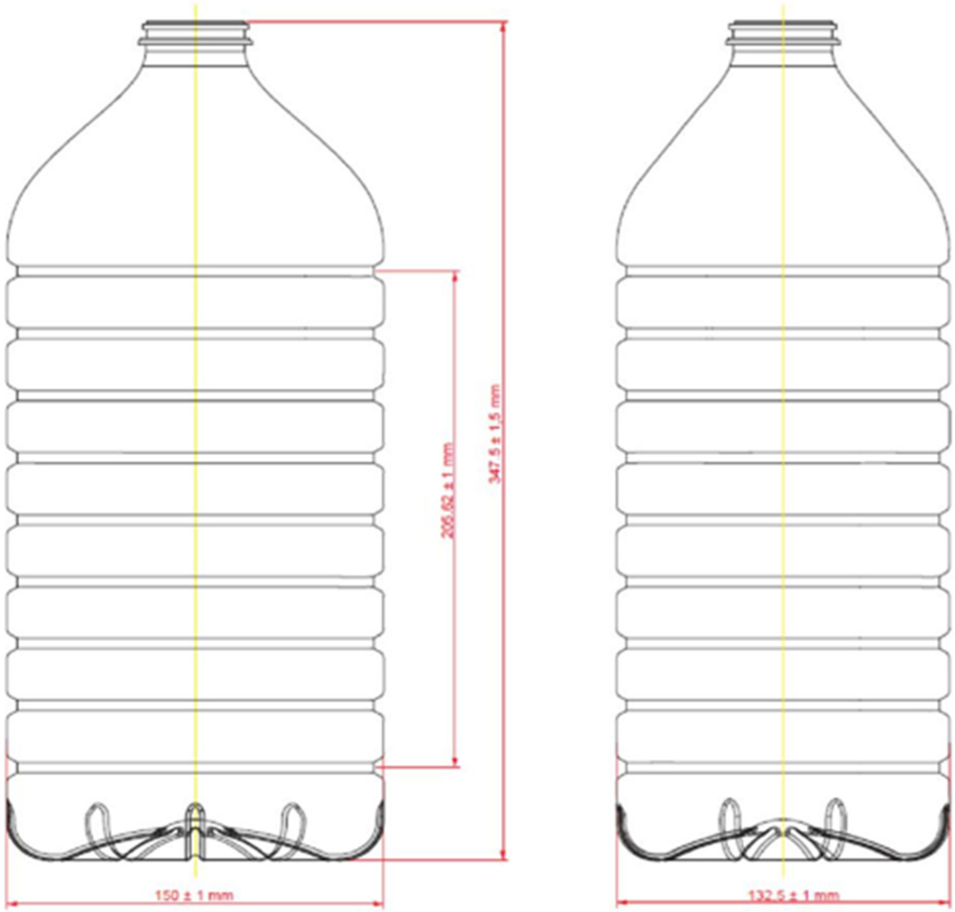


**Figure S4.** Design for 5000 mL capacity plastic bottles.

GRUPO MAEVA TORRES-MORENTE
Av. Íncar 8, CITAI
18130 Escúzar - Granada
Tel: 958 99 10 33
www.aceitesmaeva.com

# TECHNICAL DATA SHEET 5

- **SQUARE TYPE CRF PET BOTTLE 1 L**

**Table S6.** Characteristics of the 1000 mL plastic bottle known as most sale bottle.

| Attribute | Details |
|---|---|
| Material | PET |
| Filling point capacity | 1000 ± 10 mL |
| Overfill | 1050 ± 10 mm |
| Filling point | Mm |
| Weight | 23.5 ± 0.5 gr |
| Vertical load | ≥ 20 kg |
| Minimum wall thickness | 0.2 mm |
| **Dimensions** | |
| Height | 271 ± 1.5 mm |
| Width | 72.65 ± 0.5 mm |
| Depth / bottom | 72.65 ± 0.5 mm |

**Table S7.** Thickness of the 1000 mL plastic bottle known as most sale bottle.

| THICKNESS | | | |
|---|---|---|---|
| | MOLD 1 | | |
| | Measurement (mm) | SIDE A | SIDE B |
| 7 | 210 | 0,274 | 0,292 |
| 6 | 180 | 0,284 | 0,295 |
| 5 | 150 | 0,264 | 0,269 |
| 4 | 120 | 0,284 | 0,267 |
| 3 | 90 | 0,272 | 0,279 |
| 2 | 60 | 0,274 | 0,274 |
| 1 | 30 | 0,363 | 0,358 |

The color is provided through PET color additives, which are mixed with the resin during the preform manufacturing process. Both transparent and green PET have been used.

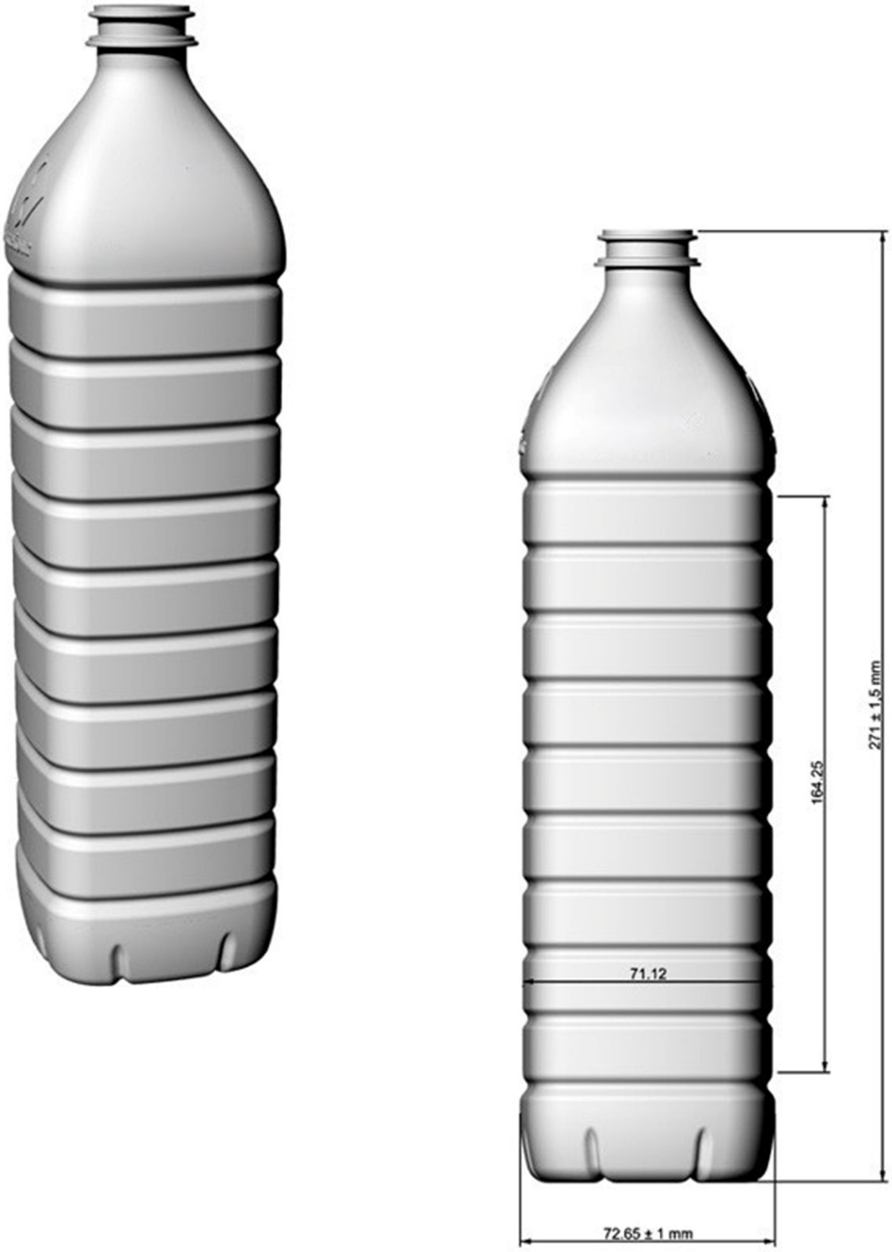


**Figure S5.** Design for 1000 mL capacity plastic bottles known as most sale bottle.

GRUPO MAEVA TORRES-MORENTE
Av. Íncar 8, CITAI
18130 Escúzar - Granada
Tel: 958 99 10 33
www.aceitesmaeva.com

# TECHNICAL DATA SHEET 6

- **SQUARE TYPE CRF PET BOTTLE 1 L**

**Table S8.** Characteristics of the 1000 mL plastic bottle.

| Attribute | Details |
|---|---|
| Material | PET |
| Filling point capacity | 1000 ± 10 mL |
| Overfill | 1050 ± 10 mm |
| Filling point | Mm |
| Weight | 36 ± 0.5 gr |
| Vertical load | ≥ 20 kg |
| Minimum wall thickness | 0.2 mm |
| **Dimensions** | |
| Height | 264 ± 1.5 mm |
| Width | 76 ± 0.5 mm |
| Depth / bottom | 76 ± 0.5 mm |

**Table S9.** Thickness of the 1000 mL plastic bottle.

| THICKNESS | | | |
|---|---|---|---|
| | MOLD 1 | | |
| | Measurement (mm) | SIDE A | SIDE B |
| 7 | 210 | 0,274 | 0,292 |
| 6 | 180 | 0,284 | 0,295 |
| 5 | 150 | 0,264 | 0,269 |
| 4 | 120 | 0,284 | 0,267 |
| 3 | 90 | 0,272 | 0,279 |
| 2 | 60 | 0,274 | 0,274 |
| 1 | 30 | 0,363 | 0,358 |

The color is provided through PET color additives, which are mixed with the resin during the preform manufacturing process. Both transparent and green PET have been used.

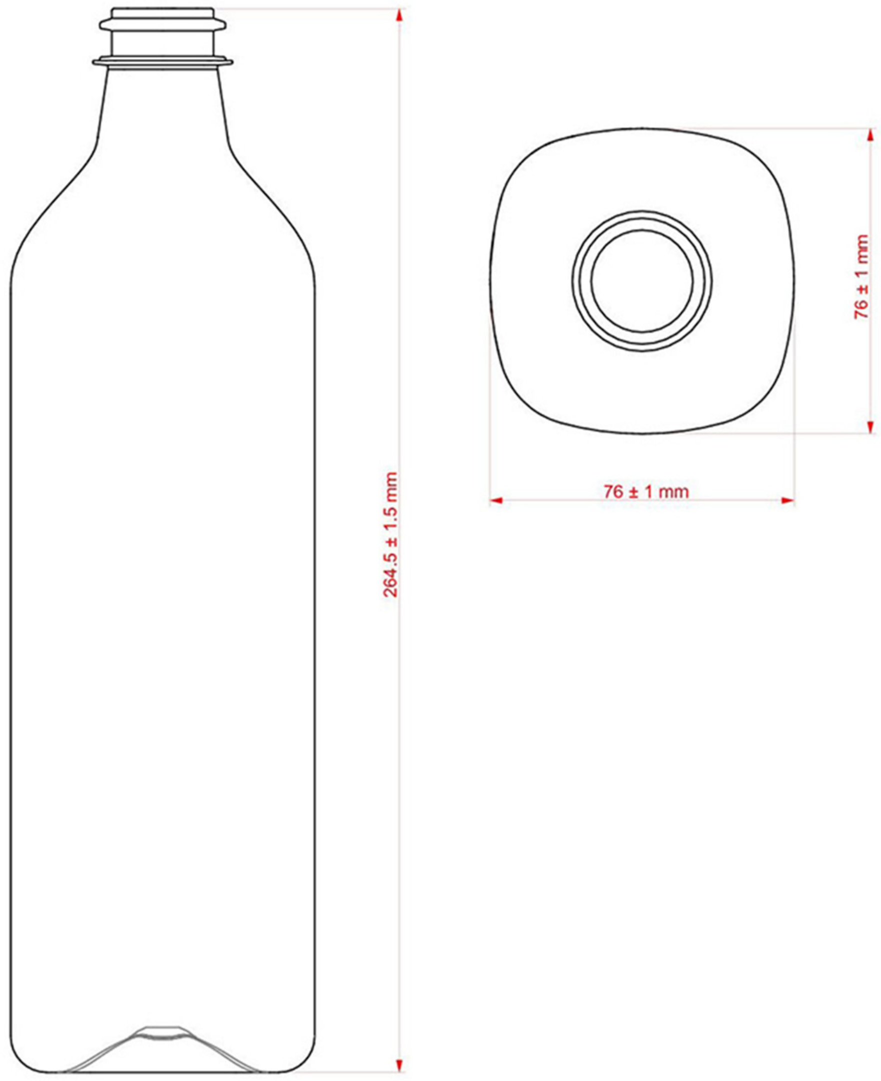


**Figure S6.** Design for 1000 mL capacity plastic bottles.

# TECHNICAL DATA SHEET 7

- **SQUARE TYPE CRF PET BOTTLE 500 mL**

**Table S10.** Characteristics of the 500 mL plastic bottle.

| Attribute | Details |
|---|---|
| Material | PET |
| Filling point capacity | 500 ± 10 mL |
| Overfill | 535 ± 10 mm |
| Filling point | Mm |
| Weight | 26.5 ± 0.5 gr |
| Vertical load | ≥ 20 kg |
| Minimum wall thickness | 0.2 mm |
| **Dimensions** | |
| Height | 196.5 ± 1.5 mm |
| Width | 65 ± 0.5 mm |
| Depth / bottom | 65 ± 0.5 mm |

**Table S11.** Thickness of the 500 mL plastic bottle.

| THICKNESS | | | |
|---|---|---|---|
| | MOLD 1 | | |
| | Measurement (mm) | SIDE A | SIDE B |
| 5 | 150 | 0,325 | 0,335 |
| 4 | 120 | 0,300 | 0,290 |
| 3 | 90 | 0,302 | 0,254 |
| 2 | 60 | 0,325 | 0,305 |
| 1 | 30 | 0,485 | 0,462 |

The color is provided through PET color additives, which are mixed with the resin during the preform manufacturing process. Both transparent and green PET have been used.

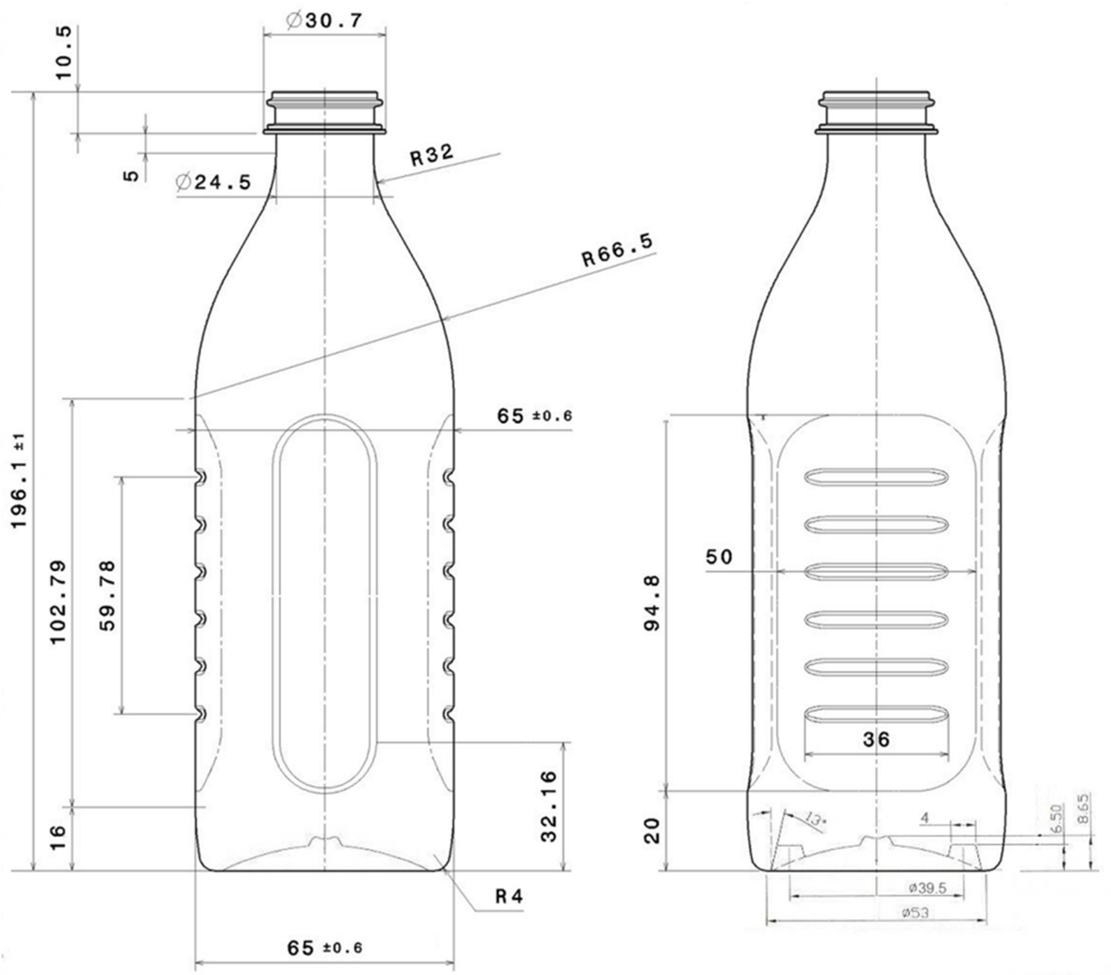


**Figure S7.** Design for 500 mL capacity plastic bottles.

GRUPO MAEVA TORRES-MORENTE
Av. Íncar 8, CITAI
18130 Escúzar - Granada
Tel: 958 99 10 33
www.aceitesmaeva.com

# TECHNICAL DATA SHEET 8

- **SQUARE TYPE CRF PET BOTTLE 250 mL**

**Table S12.** Characteristics of the 250 mL plastic bottle.

| Attribute | Details |
|---|---|
| Material | PET |
| Filling point capacity | 250 ± 10mL |
| Overfill | 275 ± 10 mm |
| Filling point | Mm |
| Weight | 22 ± 0.5 gr |
| Vertical load | ≥ 20 kg |
| Minimum wall thickness | 0.2 mm |
| **Dimensions** | |
| Height | 185.5 ± 1.5 mm |
| Width | 46.6 ± 0.5 mm |
| Depth / bottom | 46.6 ± 0.5 mm |

**Table S13.** Thickness of the 250 mL plastic bottle.

| THICKNESS | | | |
|---|---|---|---|
| | MOLD 1 | | |
| | Measurement (mm) | SIDE A | SIDE B |
| 6 | 120 | 0,381 | 0,389 |
| 5 | 100 | 0,391 | 0,401 |
| 4 | 80 | 0,569 | 0,584 |
| 3 | 60 | 0,704 | 0,729 |
| 2 | 40 | 0,719 | 0,775 |
| 1 | 20 | 0,531 | 0,569 |

The color is provided through PET color additives, which are mixed with the resin during the preform manufacturing process. Both transparent and green PET have been used.

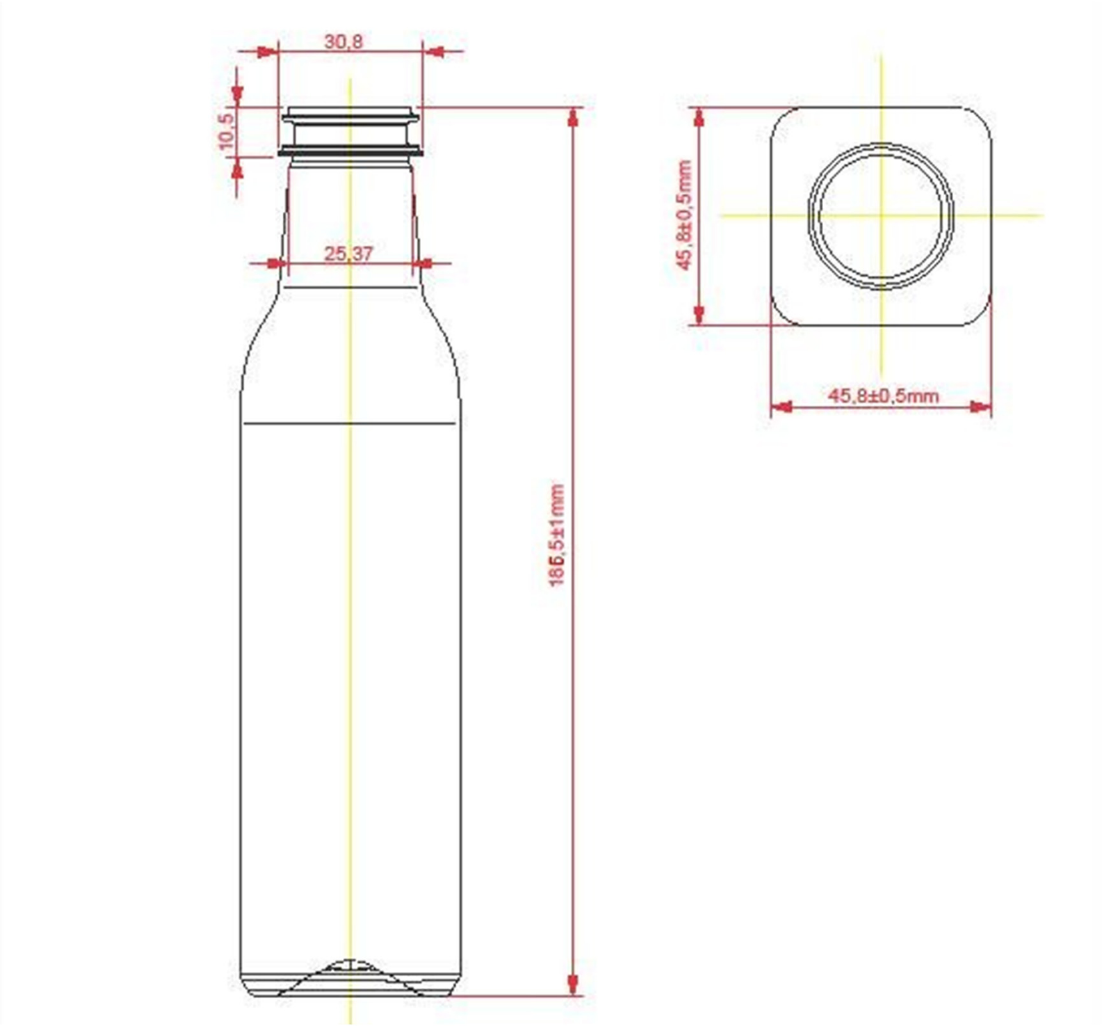


**Figure S8.** Design for 250 mL capacity plastic bottles.

**Table S14.** Average similarity index values for plastic containers.

| Bottle code | COS θ | $R^2$ | NEAR | WSI |
|---|---|---|---|---|
| PT_1000_mL[1] | 0.993 | 0.985 | **0.941** | 0.962 |
| PT_0500_mL | 0.994 | 0.987 | **0.948** | 0.966 |
| PT_0250_mL | 0.994 | 0.986 | **0.946** | 0.965 |
| PG_5000_mL[2] | 0.994 | 0.993 | **0.948** | 0.969 |
| PG_1000_mL | 0.989 | 0.976 | **0.929** | 0.952 |
| PG_1000_MS[3] | 0.994 | 0.985 | **0.943** | 0.963 |
| PG_0500_mL | 0.993 | 0.984 | **0.942** | 0.962 |
| PG_0250_mL | 0.994 | 0.986 | **0.946** | 0.965 |
| GT_0750_mL[4] | 0.994 | 0.985 | **0.943** | 0.963 |
| GT_0500_mL | 0.994 | 0.987 | **0.947** | 0.966 |
| GT_0250_mL | 0.995 | 0.988 | **0.949** | 0.968 |
| GG_0750_mL[5] | 0.978 | 0.952 | **0.900** | **0.927** |
| GG_0500_mL | 0.985 | 0.966 | **0.917** | **0.942** |
| GG_0250_mL | 0.987 | 0.971 | **0.920** | **0.945** |

[1]PT: Colourless plastic (technically transparent)
[2]PG: Green plastic
[3]MS: Most sale
[4]GT: Colourless glass
[5]GG: Green glass

Note: Values of similarity indices lower than the 0.95 threshold highlighted in bold red.

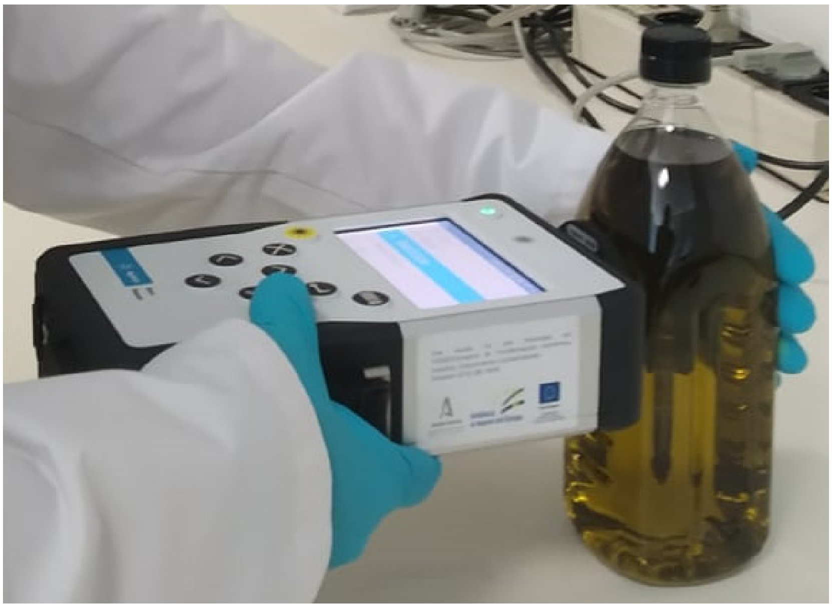

**Figure S9.** Appreciation of how the specific adapter for the Vaya Raman portable device fits onto the surface of the bottle.
